# Interlaboratory comparison of size measurements on nanoparticles using nanoparticle tracking analysis (NTA)

**DOI:** 10.1007/s11051-013-2101-8

**Published:** 2013-11-19

**Authors:** Patrick Hole, Katherine Sillence, Claire Hannell, Ciaran Manus Maguire, Matthias Roesslein, Guillaume Suarez, Sonja Capracotta, Zuzana Magdolenova, Limor Horev-Azaria, Agnieszka Dybowska, Laura Cooke, Andrea Haase, Servane Contal, Stein Manø, Antje Vennemann, Jeans-Jacques Sauvain, Kieran Crosbie Staunton, Sergio Anguissola, Andreas Luch, Maria Dusinska, Rafi Korenstein, Arno C. Gutleb, Martin Wiemann, Adriele Prina-Mello, Michael Riediker, Peter Wick

**Affiliations:** 1 NanoSight Ltd., Minton Park, London Road, Amesbury, Wiltshire, SP4 7RT UK; 2 Trinity Centre for Health Sciences, Department of Clinical Medicine, Institute of Molecular Medicine, St. James’s Hospital, James Street, Dublin 8, Ireland; 3 Laboratory for Materials - Biology Interactions, Swiss Federal Laboratories for Materials Research and Testing (Empa), Lerchenfeldstrasse 5, 9014 St Gallen, Switzerland; 4 Institute for Work and Health, Rte. de la Corniche 2, 1066 Epalinges-Lausanne, Switzerland; 5 NanoSight USA, 1415 Washington Heights, Rm 6611, Ann Arbor, MI 48109 USA; 6 Health Effects Laboratory, Department of Environmental Chemistry, Norwegian Institute for Air Research, Instituttveien 18, P.O. Box 100, NO-2027 Kjeller, Norway; 7 Department of Physiology and Pharmacology, Sackler School of Medicine, Tel Aviv University, 69978 Tel Aviv, Israel; 8 Department of Earth Sciences, Natural History Museum, Cromwell Road, London, SW7 5BD UK; 9 Centre for Bio-Nano Interactions (CBNI), University College Dublin, Belfield, Dublin 4, Ireland; 10 Experimental Research, Department of Product Safety, Bundesinstitut fur Risikobewertung (BfR), Max-Dohrn-Strasse 8-10, 10589 Berlin, Germany; 11 Département Environnement et Agro-biotechnologies (EVA), Centre de Recherche Public Gabriel Lippmann, 41, rue du Brill, 4422 Belvaux, Luxembourg; 12 IBE R&D Institute for Lung Health gGmbH, Mendelstrasse 11, 48149 Münster, Germany

**Keywords:** Nanoparticle, Interlaboratory comparison, Reproducibility, Polydispersity, Toxicology, Health and safety implications

## Abstract

One of the key challenges in the field of nanoparticle (NP) analysis is in producing reliable and reproducible characterisation data for nanomaterials. This study looks at the reproducibility using a relatively new, but rapidly adopted, technique, Nanoparticle Tracking Analysis (NTA) on a range of particle sizes and materials in several different media. It describes the protocol development and presents both the data and analysis of results obtained from 12 laboratories, mostly based in Europe, who are primarily QualityNano members. QualityNano is an EU FP7 funded Research Infrastructure that integrates 28 European analytical and experimental facilities in nanotechnology, medicine and natural sciences with the goal of developing and implementing best practice and quality in all aspects of nanosafety assessment. This study looks at both the development of the protocol and how this leads to highly reproducible results amongst participants. In this study, the parameter being measured is the modal particle size.

## Introduction

Nanotechnology is rapidly developing new applications and advanced materials into many manufacturing areas from information technology, energy storage and harvesting, to radically new medical technologies. The projected market figure for nanotechnology incorporated in manufactured goods by 2020 is approximately 3,000 billion US dollars worldwide (Roco 2011). Such exponential global growth is, however, calling for responsible and quantitative evaluation of the development of manufacturing nanomaterials and its associated metrology. This is particularly true since nanomaterials have unique physical and chemical properties that are useful for various consumer and industrial applications, but these very same properties may give rise to unique biological reactivity. This has led to mounting concerns over the safety of nanomaterials and pressure to control the potential risks (Schrurs and Lison 2012). To ensure compliance with environmental protection guidelines (OECD 2009) nanoparticles (NPs) produced, either directly or indirectly, must be fully characterised (Hassellöv et al. 2008; Tiede et al. 2009). This is fundamental in all areas of research and industry.

Among the different properties which need full characterisation, the size of NPs and the quality and stability of their dispersion often have a profound effect on their interactions with organisms and the environment. It has been extensively reported that NP response to the surrounding environment is size dependent (Jiang et al. 2008; Tenzer et al. 2011) due to their large surface area that interacts with their surrounding matrix, and this can influence their reactivity with toxicity targets in the cells (Lison and Huaux 2011; Tsao et al. 2011). This has been found to be particularly relevant when the NPs are used for targeted applications. For instance, gold NPs have been used in medical application as contrast agents or nanocarriers (Tong et al. 2009; Kim et al. 2010), polystyrene NPs are a good model for diagnostics and environmental applications due to their relatively well-defined size and low cost (De Jong and Borm 2008; Fritz et al. 1997), silica NPs as drug delivery carriers due to their size-dependent toxicity (Greish et al. 2012; Lin and Haynes 2010; Mohamed et al. 2012) and iron oxide nanoparticles (SPIONS) as therapeutics carriers (Prina-Mello et al. 2013). Therefore, when working with NPs, it becomes crucially important to fully characterise the NPs physico-chemical properties and their interaction with the surrounding matrix or environment (Montes-Burgos et al. 2010; Warheit 2008). This is particularly true when investigating the efficacy and biodistribution of NPs in vivo where it is extremely difficult to validate any mechanism of interactions or kinetics from the biodistribution data without having accurate particle size distributions (Gaumet et al. 2008). Therefore the dispersion state of the NPs in solution becomes a main parameter to be investigated which can be correlated to particle stability, and subsequent shelf life and efficacy (Hassellöv et al. 2008). Thus, the implications are not only limited to biological applications but also to environmental and ecological perspectives, as well as regulatory (Elsaesser and Howard 2012; Hanna et al. 2013).

In fact, it has been extensively reported by many studies that the nature of the media in which the NPs are dispersed is driving their response and interaction at a biological level (Lison and Huaux 2011; Schrurs and Lison 2012). Several reports have defined and characterised some aspects of this bio–nano interaction through association with the NP physiochemical properties (Nel et al. 2009); particle size being the starting aspect for aggregation consideration, formation of protein coronas (Casals et al. 2010; Cedervall et al. 2007) and subsequent shielding of targeting moieties resulting in loss of NP specificity (Salvati et al. 2013).

Several techniques have been developed for measuring particle size, shape and dispersity from a suspension of particles; electron microscopy (EM), dynamic light scattering (DLS), disc centrifugation, Coulter principle and NTA amongst others. Some of these are labour intensive and time demanding whereas others are cost effective and user friendly. The most frequently used and user friendly NP size characterisation technique is DLS (Filipe et al. 2010). This technique measures the fluctuations in scattered light intensity caused by NPs moving under Brownian motion (Frisken 2001). However, the analysis is weighed towards larger particle size, and as a result, the presence of NP aggregates will bias the particle size distribution, resulting in inaccurate size determination. The use of NTA, with a lower concentration detection limit compared to DLS, analysing NPs on a particle by particle basis, offers a new method for the visualisation and characterisation of NPs in suspension.

The QualityNano project is a European Union Framework Programme 7 funded infrastructure project for developing best practice and innovation in nanomaterial safety testing. One fundamental activity of the QualityNano project is the establishment of quality control and quality assurance conditions for nanomaterial safety and assessment. As technologies and methodologies develop in this area, and the number of end-users increases, the validity of the methodologies and standards needs to be continuously monitored and updated to the most stringent industrial requirements. Quality standards can only be achieved by enlarging statistical numbering and introducing multivariate analysis. This can be achieved by adopting a systematic approach to parametrically assess the interlaboratory, inter-batches and multi-users variation. NTA has only been commercialised since 2006 (Filipe et al. 2010) but the technique has, in recent years, grown rapidly in its adoption and use, with over 600 systems and 800 third party papers, consolidating the technique across many areas of application, such as therapeutic NP characterisation, developing solutions for diagnostics (e.g., exosomes), drug delivery and cancer treatment, development of bioanalytical assays, vaccine characterisation and nanotoxicology. Therefore the adoption of an interlaboratory comparison (ILC) to validate the NTA technique, by assessment of panels of nanomaterials from within the QualityNano project, derives from the extensive knowledge accumulated and shared between the QualityNano partners and NanoSight Ltd., Amesbury, UK, the company that manufactures the NTA systems. NTA performance is therefore assessed by a structured ILC using defined samples measured by multiple laboratories in order to ascertain the competence of the laboratory, the quality of the standard operating procedures (SOPs) and the reproducibility of the technique. Furthermore, through the use of defined protocols for measurement of standardised samples, the ability of the different NTA systems under evaluation to produce reliable and reproducible results can be investigated (multi-user’s variation). It is therefore important to highlight that the uncertainty in the validity and consistency of results could have important implications for the determination of the effects of NPs since different results could be generated when applying different starting dispersions, thus confusing the outcomes. During NP characterisation, using best practice in-house developed SOPs does not always guarantee consistency with other laboratories, as previously shown in Roebben’s study for the DLS technique (Roebben et al. 2011). The recommendation for adopting an ILC study is therefore ideal for both developing and testing protocols, as demonstrated in the study presented here, which has been strongly supported within the QualityNano consortium by NanoSight. The authors in this manuscript adopted the ILC principles for assessment from Roebben and coworkers and transferred this to the NTA technique. For instance, this study adopted the use of National Institute of Standards and Technology (NIST) traceable NPs and the supply and modification of protocols following each analysis round. Reproducibility analysis was carried out using classical statistical analysis based around the arithmetic mean values using the ISO 5725-2 approach (ISO 1994) and robust statistical analysis centred around methods described in ISO 5725-5 (ISO 1998). The ILC was also assessed in its inter-batch and multi-users variation carried out over four rounds using blind samples and well-defined SOPs to produce a protocol for NP size characterisation using NTA in order to reinforce the robustness of the NTA technique, its standardisation and effort towards defining regulatory guidelines.

### NTA: technique principle and details

**Fig. 1:**
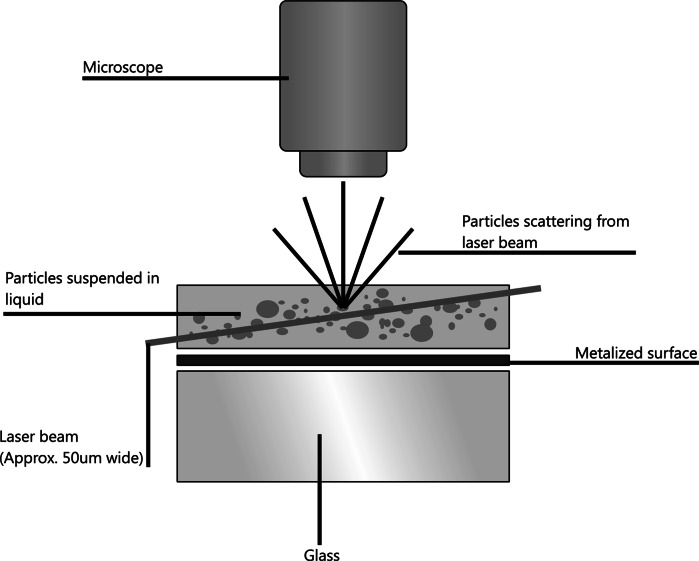
Schematic of the optical configuration used in NTA

NTA utilises the properties of both light scattering and Brownian motion in order to obtain particle size distributions of samples in liquid suspension. A laser beam (of arbitrary wavelength but typically those available from laser diodes operating at 635, 532, 488, or 405 nm) is passed through a prism-edged glass flat within the sample chamber. The angle of incidence and refractive index of the glass flat are designed to be such that when the laser reaches the interface between the glass and the liquid sample layer above it the beam refracts, resulting in a compressed beam with a reduced profile and high power density. The particles in suspension in the path of this beam scatter light in such a manner that they can be easily visualised via a long working distance, 20× magnification microscope objective, fitted to an otherwise conventional optical microscope. Onto this is mounted either a charged coupled device (CCD), electron multiplied charged coupled device (EMCCD) or high-sensitivity complementary metal–oxide–semiconductor (CMOS) camera, operating at approximately 30 frames per second (fps), which captures a video file of the light scattered by particles moving under Brownian motion, within a field of view of approximately 100 μm × 80 μm × 10 μm (Fig. 1).

Within the field of view, particles are seen moving under Brownian motion, either directly by eye using the microscope oculars or via the camera. The proprietary NTA software records a video file (of typically 30–60 s duration) of the particles viewed and then simultaneously identifies and tracks the centre of each particle on a frame-by-frame basis. The image analysis software then determines the average distance moved by each particle in the x and y directions. This value allows the particle diffusion coefficient (*D*_t_) to be determined, from which, if the sample temperature (*T*) and solvent viscosity (*η*) are known, then the sphere-equivalent hydrodynamic diameter (*d*) of the particles can be identified using the Stokes–Einstein equation (Eq. 1).

1 $$D_{\text{t}} = \frac{{TK_{\text{B}} }}{3\pi \eta d}$$

where *K*_B_ is Boltzmann’s Constant.

Brownian motion occurs in three dimensions but NTA observes motion only in two dimensions. However, the fact that three-dimensional Brownian motion is tracked only in two dimensions is accounted for by the use of the following variation of the Stokes–Einstein equation (Eq. 2). It is possible, however, to determine *D*_t_ from measuring the mean squared displacement of a particle in one, two or three dimensions (Eq. 2, respectively).

2 $$\mathop {\overline{{(x^{2} )}} = \frac{{2TK_{B} t}}{3\Uppi \eta d}}\limits_{\text{a}} \quad \quad \mathop {\overline{{(x,y)^{2} }} = \frac{{4TK_{B} t}}{3\Uppi \eta d}}\limits_{\text{b}} \quad \quad \mathop {\overline{{(x,y,z)^{2} }} = \frac{{2TK_{B} t}}{\Uppi \eta d}}\limits_{\text{c}}$$

Thus, in the case where measurement of movement in two dimensions is made; Eq. 2b is employed.

One crucial advantage that NTA has over other measurement techniques is that it is not biased towards larger particles or aggregates. The software is based on the tracking of single particles, whereas typical DLS techniques place a strong bias on the largest particles present in the sample (Filipe et al. 2010). NTA therefore allows for the detection of secondary peaks, which may not be detectable or resolvable using other traditional measurements.

### NTA: standardisation

Early steps towards standardisation of NTA have recently been demonstrated by an ASTM guidance paper published about the technique (ASTM 2012) providing an overview of the methodology to be followed for good practice and discussing aspects such as principles and limitations of the method, considerations of sampling, necessary concentration and interpretation of results, with particular reference to comparison with other techniques. An International Standardisation Community at ISO TC24 welcomed the submission of a new work item proposal on Particle Tracking Analysis (PTA)/NTA at their recent meeting (ISO 2013).

Standardisation of the NTA technique has previously been difficult to achieve due to the number of software options that a user has been required to choose in order to make a measurement. This was highlighted in an excellent paper comparing DLS and NTA (Filipe et al. 2010) which highlighted this issue of subjectivity of NTA results with user settings. Table 1 summarises the development of the software since that point, showing the progression of algorithm development allowing these settings to be effectively automated.

**Table 1:** History of development of NTA software showing list of parameters along with the number of user adjustable settings for each parameter

Part of analysis	Year	2009	2010	2011	2012
	NTA Version	2.0	2.1	2.2	2.3
Data collection	Capture time	215	215	5	5
	Shutter	1500	1500	1	1
	Camera gain	680	680	16	16
	Gamma	2	1	1	1
Data analysis	Brightness	186	186	1	1
	Image gain	1000	1000	1	1
	Blur	5	4	1	1
	Detection threshold	188	100	50	20
	Max blob size	3000	1	1	1
	Min track length	183	1	1	1
	Min expected particle size	9	9	4	1
	# Free user setting	11	8	4	3
	# Variations	3.8 × 10^23^	1.5 × 10^17^	1.6 × 10^4^	1.6 × 10^3^

The ILC assessment design in this study employs the NTA2.3 software version which had three settings that users should set. These were: (1) capture time (unified for all laboratories and users by supplied SOP), (2) camera level and (3) detection threshold (guidance provided for 2 and 3 by the supplied SOP).

### NTA: regulatory aspect

It is expected that the outcomes of ILC studies of the NTA technique could contribute to the defining of regulatory policy and guidelines for the adoption of safe nanotechnology procedures in line with the goals of QualityNano, through the generation of SOPs for the accurate and reproducible size characterisation of NPs dispersed in water-based solvents.

## Materials and methods

### Test materials

NIST traceable polystyrene nanospheres, nominally 100 and 200 nm (1 % solids), were purchased from Thermo Fisher Scientific Inc., whilst gold NPs, nominally 60 and 80 nm (0.01 % solids), were purchased from BB International, Cardiff. Ham’s F10 nutrient mix and Bovine Serum Albumin (BSA) were purchased from Sigma Aldrich Co. HPLC grade water was purchased from Rathburn Chemicals Ltd., Scotland. 0.02 μm Anotop 25 syringe filters (Whatman GmbH, Germany) were used to filter water and BSA samples prior to analysis.

### Samples preparation

Samples were aliquoted from a single lot prior to the start of each round (either pre-diluted or neat), ensuring all participating labs received aliquots of the same materials. Rounds 1 and 2 were carried out on the same four monodisperse samples, dispersed in water, round 3 analysed three monodisperse samples, again dispersed in water. Round 4 included a bimodal sample, and monodisperse nanospheres dispersed in biological media with and without the addition of BSA. The details of the composition and characteristics of the samples used in the present study are summarised in Table 2.

**Table 2:** Characteristics of nanoparticle samples used in the study

Particle composition	Source	Round robin	Nominal size (nm)	Initial concentration (% solids)	Diluent
Gold	BBI	1 and 2	30 ± 2 (TEM)	~0.01	H_2_O
Carboxylated polystyrene	Invitrogen	1 and 2	100 ± 11 (TEM)	~0.1	H_2_O
Aminated polystyrene	Polysciences	1 and 2	100	~0.1	H_2_O
Silica	Polysciences	1 and 2	100	~0.1	H_2_O
Polystyrene	Thermo Scientific	3	102 ± 3 (TEM)	~1	H_2_O
Gold	BBI	3	60 ± 3 (TEM)	~0.01	H_2_O
Gold	BBI	3	81 ± 4 (TEM)	~0.01	H_2_O
Polystyrene	Thermo Scientific	4	102 ± 3 (TEM)	~1	H_2_O, Ham’s F10 Nutrient Mix, BSA
Polystyrene	Thermo Scientific	4	203 ± 5 (TEM)	~1	H_2_O
Gold	BBI	4	81 ± 4 (TEM)	~0.01	H_2_O

All samples were distributed from NanoSight. Samples for rounds 1 and 2 were prepared by University College Dublin, samples for rounds 3 and 4 were prepared by NanoSight. Protocols were all developed and distributed by NanoSight. Effort was taken to ensure samples were always analysed blind by participants, however, the solvent and the material were known, for MSDSs to be distributed.

### Participants and systems

Twelve Laboratories from Europe and the USA participated in the four rounds of the ILC using instruments from NanoSight Ltd., Amesbury, UK, between September 2012 and April 2013 as per manuscript authorship. Laboratories were each allocated a unique participant number allowing results to remain anonymous. The participants in this comparison were not pre-screened in any way, some users having received no direct training on the system in their laboratory. The key characteristics of the group were that they were eager to ensure that they were using their systems correctly and to acquire reproducible results. Participants were given no additional training, although where issues/outlying results were identified, they were investigated thoroughly to establish the root cause, the participants informed and the protocol appropriately improved (where possible).

Details of the NanoSight platforms, laser wavelengths and camera type used by each laboratory are given in Table 3. Laboratories reported results in the form of summary files (output by NTA) for each sample. Results were collated and analysed by NanoSight.

**Table 3:** NanoSight systems used for ILC by participant number

Lab code	Platform	Camera	Laser wavelength (nm)	Temperature control
1	LM10	sCMOS	532	Yes
2	NS500	EMCCD	532	Yes
3	NS500	sCMOS	405	Yes
4	NS500	sCMOS	405	Yes
5	LM20	CCD	635	No
6	LM10	CCD	635	No
7	LM10	CCD	635	No
8	LM20	CCD	635	No
9	NS500	sCMOS	405	Yes
10	LM20	CCD	635	No
11	LM10	EMCCD	405	Yes
12	LM10	sCMOS	405	No

### Protocol Development

Results were disseminated to all partners following rounds 2, 3 and 4. This allowed discussion with partners about how to proceed and allowed the group’s expertise to be employed. Choice of samples for a round was only made following the collection and analysis of the results from the preceding round. This allowed an assessment of the progress, the choice of appropriate samples to challenge progress and time for development/improvement of the protocol.

#### ILC round 1

A preliminary ILC (round 1) was performed on 4 samples (nominally gold 30 nm, 100 nm carboxylated polystyrene, 100 nm aminated polystyrene and 100 nm silica), without any protocol provided. Participants analysed the samples according to their own ‘in-house’ protocols for using NTA. This highlighted a number of issues, such as the need to perform replicate measurements on each sample and a need to ensure sample dilution to an appropriate concentration for the NTA system, which were incorporated into the protocol for subsequent rounds.

#### ILC round 2

Round 2 analysed the same samples as round 1, but under a protocol developed to standardise methods between participating laboratories. The protocol covered sample handling and storage, sample preparation (standard dilutions for each sample), video capture (60 s duration) and data analysis and export. Each laboratory analysed each sample in triplicate to allow statistical analysis under repeatability conditions.

#### ILC round 3

The results and particle sizes from rounds 1 and 2 being known by the participants, three new samples were supplied for analysis in round 3 (nominally 100 nm polystyrene, 60 nm gold and 80 nm gold), along with an updated protocol, which was amended in light of the results for round 2. Samples were shipped with a temperature sensor which indicated if the contents were exposed to temperatures below 4 °C. Laboratories were also supplied with a range of disposables, including 0.02 μm syringe filters and HPLC grade water. Updates to the protocol included a system recalibration step (if required), software update to the newest version of NTA, vibration checks (to prevent mis-sizing), and recording six replicate videos for each sample to improve statistical analysis.

#### ILC round 4

Unlike previous rounds, where all the samples were monodisperse nanospheres diluted in water, round 4 investigated both a bimodal sample (80 nm gold and 200 nm polystyrene) and monodisperse nanospheres (100 nm polystyrene) suspended in biological media (Ham’s F10 nutrient mix) and biological media supplemented with BSA (Ham’s F10 nutrient mix plus 5 mg/ml BSA). This allowed analysis of samples in more complex aqueous solutions than water. Samples were shipped with two temperature sensors, to indicate if the contents were exposed to temperatures below 4 °C or above 29 °C.

## Results and discussion

Laboratories submitted reports containing modal sizes for each replicate of each sample run, along with the particle size distributions, with outlying results being discarded [consisting of 0 (of 48), 6 (of 48), 0 (of 36) and 3 (of 44) from rounds 1, 2, 3 and 4, respectively]. During each of the rounds where technical causes could be unambiguously identified or it was ascertained that a participant was not fully complying with the protocol, these results were discarded. This was (usually) assumed to be a weakness of the protocol, which was then modified to better ensure compliance in future ILC rounds.

Method reproducibility analysis was carried out using methods laid out in ISO 5725-2 (ISO 1994). The modal sizes obtained for each sample from rounds 2, 3 and 4 were analysed using both classical [average and standard deviation (SD) based] and robust [median and median absolute deviation based (MAD)] statistics as per ISO 5725-5 (ISO 1998). Robust statistics has the advantage that it is much less sensitive to outliers and so a median value better represents the centre of a distribution of results reported from different laboratories. In addition, the SD describing a second order effect is considerable more sensitive towards results that are further away from the mean value, and are therefore potential outliers, than towards all the results that are close to the mean value. MAD, as median absolute deviation, is less sensitive to large deviations at the extremes.

**Fig. 2:**
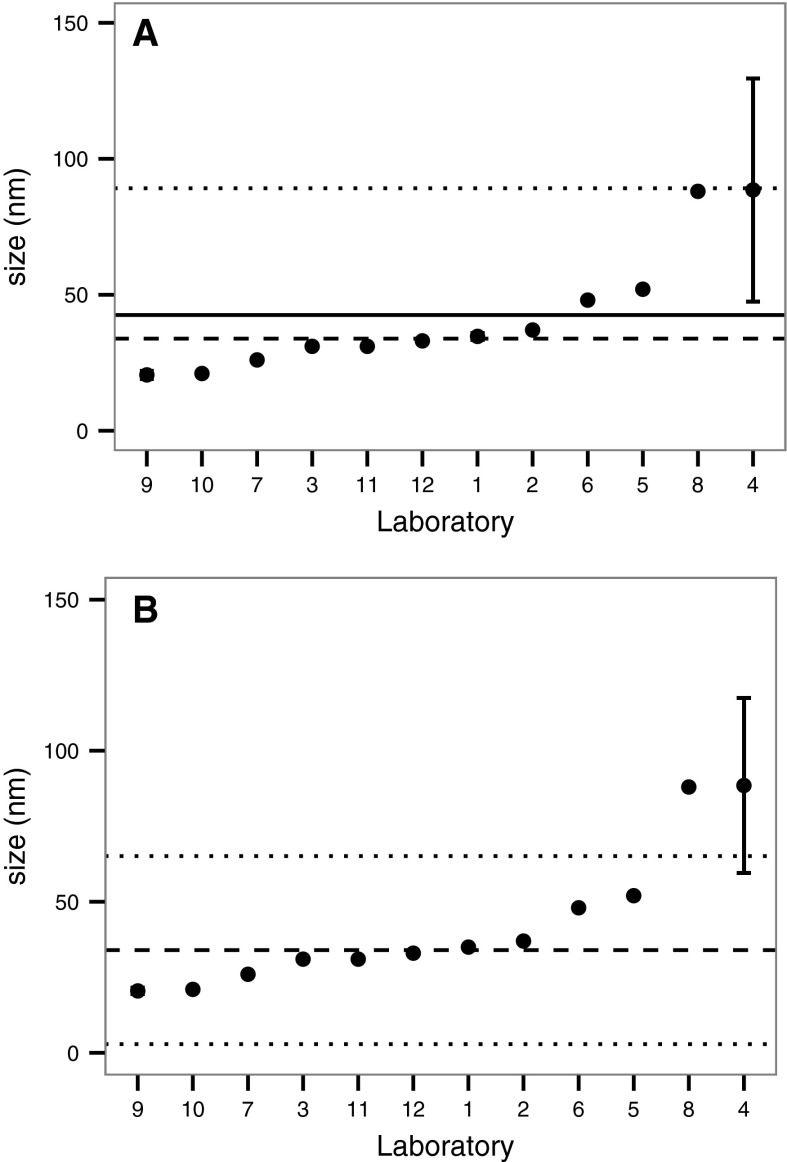
Modal particle size for 30 nm gold particles from ILC round 1 showing results from each partner ordered by increasing modal value based on **a** classical (±2 SD) and **b** robust (±2 MAD) statistical analysis. The mean value (*solid line*), median (*dashed line*) and 95 % confidence intervals (*dotted line*) are also shown

Analysis of the results obtained from ILC round 1 shows the variation and lack of reproducibility of results from the 12 laboratories when no protocol was supplied. Figure 2 summarises the modal size results obtained during ILC round 1 on 30 nm gold nanospheres based on standard (a) and robust (b) statistical analysis. It appears clear from Fig. 2 that results from 2 of 12 laboratories do not match with the data of the other 10 partners. Therefore, in the case of classic statistical analysis the mean value is biased due to the presence of these outlier results (Fig. 2a) whereas most of the values are close to the median value. As expected, this discrepancy between mean and median values is considerably reduced by the robust statistical data treatment, which is typically more appropriate for such a study (Fig. 2b). The range of the 95 % confidence interval is also smaller. This poor reproducibility of an interlaboratory test, as indicated by a coefficient of variation of 54.8 %, can easily be attributed to the absence of guidance in the ILC round 1 which included users who had had no training (either internal or external) in the technique or on their system. The lack of an adequately defined measurand in ILC round 1 resulted in an excessive uncertainty of the measurement, as predicted by the guide to the uncertainty in measurement (GUM) (JGCM 2008).

Between the last 3 rounds, the protocols were upgraded on the basis of issues encountered in the preceding round, covering all the aspects of the experimental process: sample shipping (temperature sensors), sample handling and preparation (e.g., dilution), sample storage, control of measurement parameters (e.g., video control, detection threshold), software update (e.g., vibration check) and analytical data treatment (e.g., number of replicates, statistic models).

**Fig. 3:**
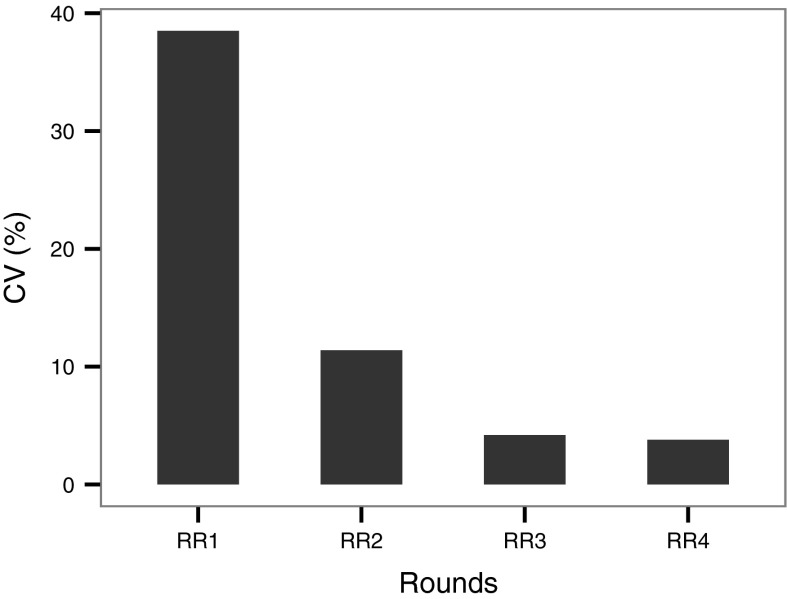
Evolution of the average percentage coefficient of variation from ILC round 1 to ILC round 4. The percentage coefficient of variation is shown to decrease with each round due to improvements within the protocol

The positive impacts that a well-established protocol and good compliance exert on the robustness of the NP size determination with NTA technique is further emphasised when we consider the calculated coefficient of variation as a comparative parameter between the different ILC rounds. The percentage coefficients of variation are summarised in Table 4 showing a clear link between the improving reproducibility of measurements and successive rounds. The averaged percentage coefficient of variation calculated for monodisperse samples progressively decreases from ILC round 1 to round 4: 38.5, 11.4, 4.2 and 3.8 % (Fig. 3).

**Table 4:** Percentage coefficient of variation for each sample in the ILC

ILC Round	1 (% CV)	2 (% CV)	3 (% CV)	4 (% CV)
30 nm gold	54.8	10.5		
100 nm carboxylate polystyrene	33.3	9.3		
100 nm aminated polystyrene	31.2	15.9		
100 nm silica	34.5	10.0		
100 nm polystyrene			3.5	3.1
60 nm gold			5.1	
80 nm gold			4.1	
100 nm polystyrene + nutrient Mix				3.7
100 nm polystyrene + nutrient mix + BSA				4.7
80 nm gold (in bimodal sample)				5.5
200 nm polystyrene (in bimodal sample)				5.2
Average (% CV)	38.5	11.4	4.2	4.4
				3.8^a^

^a^Considering only monodisperse samples

Once again, the combination of a powerful analytical technique with improved protocols gave rise to robust and reproducible determination of the modal size of both monodisperse and bimodal nanosphere samples prepared in water and complex biological matrices.

**Fig. 4:**
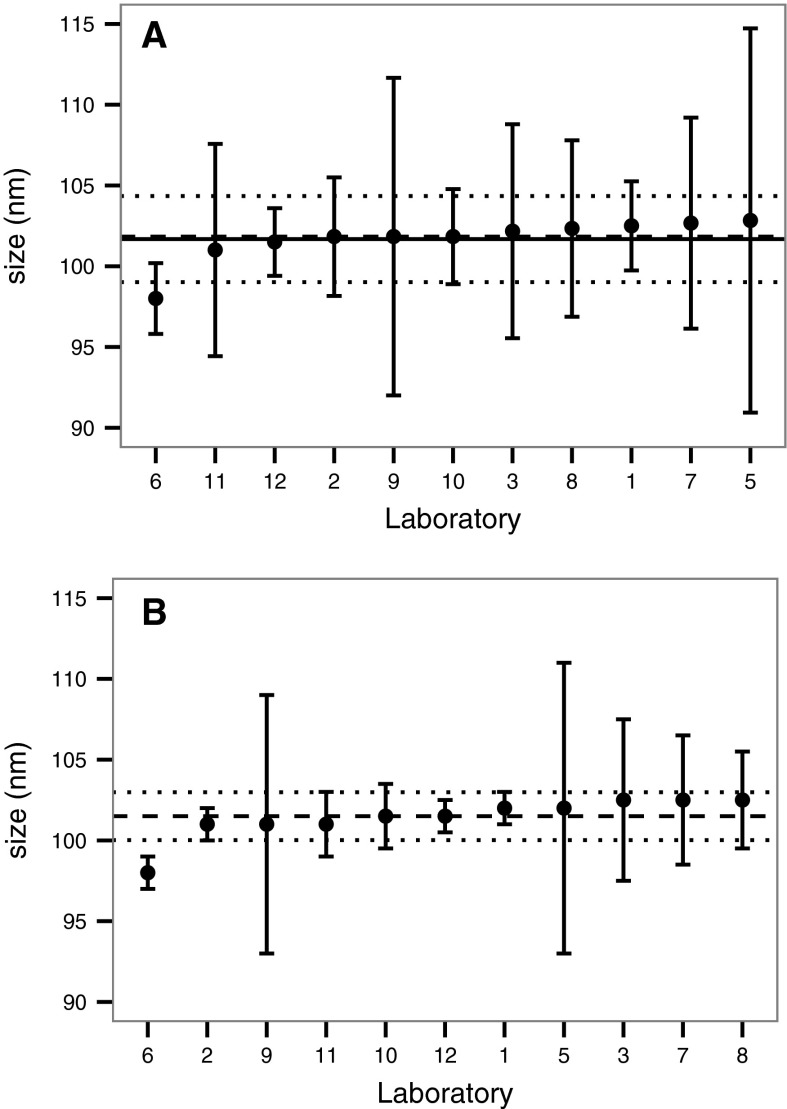
Modal particle size for 100 nm polystyrene particles from ILC round 4, showing results from each partner ordered by increasing modal value based on **a** classical (±2 SD) and **b** robust (±2 MAD) statistical analysis. Mean (*solid line*), median (*dashed line*) and 95 % confidence intervals (*dotted line*) are also shown

Standard and robust analyses were established with participant results from all ILC rounds. In the case of ILC round 4, the modal size results obtained on a nominal 100 nm polystyrene nanospheres based on classical and robust statistics are reported in Fig. 4a and b, respectively. In that round, the results obtained for 10 laboratories out of a total of 11 are within the 95 % confidence interval and so match with the mean and median values, for both classical and robust statistical analyses. The range of the 95 % confidence interval is dramatically decreased in comparison to the one obtained in ILC round 1. As mentioned earlier, the very low coefficient of variation calculated at ILC round 4 for monodisperse solution of polystyrene nanosphere (3.1 %) strongly emphasises the effect of a well-established SOP for highly reproducible and accurate NTA measurements.

**Fig. 5:**
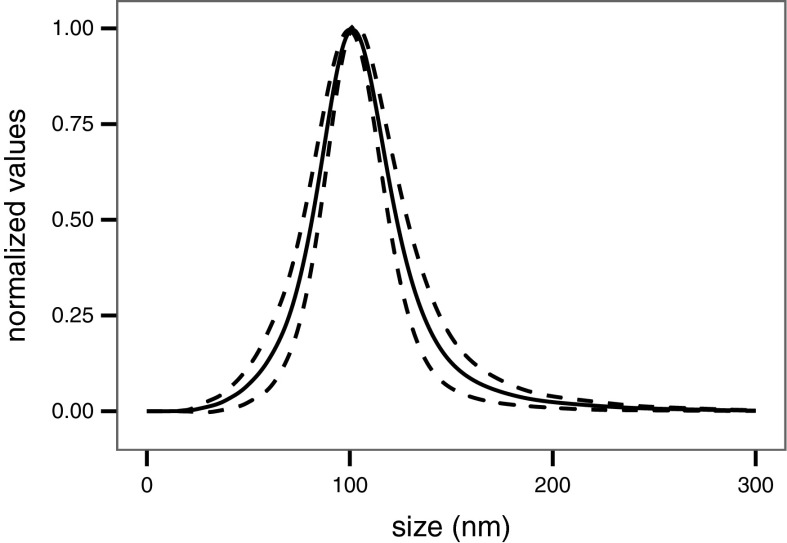
Averaged particle size distribution of nominally 100 nm polystyrene nanospheres in water analysed by 11 laboratories in ILC round 4. Size distribution ±1 SD is represented by the* two dashed lines*

**Fig. 6:**
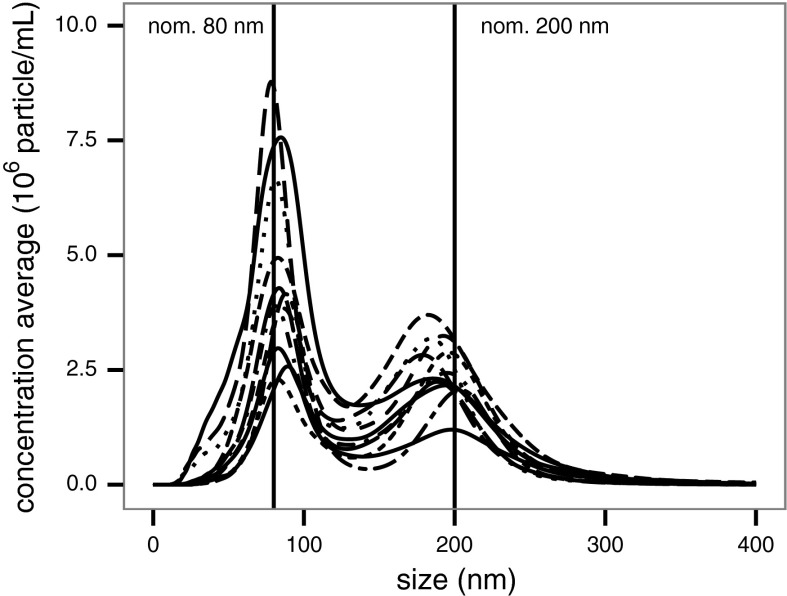
Averaged particle size distributions of a bimodal mix of nominally 80 nm gold and nominally 200 nm polystyrene nanospheres in water analysed by 11 laboratories in ILC round 4

By ILC round 4, the averaged modal particle size distribution resulting from the analysis of 11 laboratories (one laboratory failed to supply data) on monodisperse polystyrene nanospheres (100 nm) diluted in water clearly demonstrates the analytical reliability and power of the NTA technique, when supported by a well-defined SOP, as shown in Fig. 5. The average modal size and coefficient of variation corresponding to this set of measurements were calculated to be 101.7 nm and 3.12 %, respectively. Similarly, during the same round bimodal samples containing a mixture of nominally 80 nm gold (77–85 nm, TEM) and 200 nm polystyrene (203 ± 5 nm, TEM) nanospheres were also analysed and the modal particle size distributions obtained by 11 laboratories are depicted in Fig. 6. In that case, the calculated average modal sizes were 84.0 and 190.7 nm with corresponding coefficients of variations of 5.47 and 5.16 %, respectively. Both the relatively high NP size measurement accuracy and reproducibility confirm that NTA technique is also well adapted for the size analysis of bimodal samples.

## Conclusion and outlook

Overall, it is clear that in the absence of shared guidelines on how to use a system and prepare samples, variability across laboratories can be large even for relatively monodispersed samples (ILC1). The definition of a shared protocol allowed improved results to be obtained (ILC2), however, the ILC exercise clearly outlines that even a well-defined protocol (much more complete and detailed than what is usually described in the experimental section of published work) can still lead to a (~4 % CV) variability of outcomes. This has clear implications for the on-going debate on the definition of guidelines for reporting research in this field.

The ILC has shown that, with the guidance of a well-written protocol, users of NTA (even those with little or no training on the instrument) are able to obtain reproducible and accurate modal particle size results on a range of samples, both monodisperse and bimodal, on samples dispersed in water or biological media and on particles of different sizes and material composition. It is important to note here that the samples used in this study were all nominally spherical.

This study has shown that a well-designed and focused series of round robins can help identify relevant issues that have a significant effect on measurement results. It has been demonstrated to be an effective way of improving the understanding of the basics of measurement techniques such as NTA. This leads to a better specification of the measurand, and therefore to a more elaborate and more useful protocol, detailing important steps in the measurement procedure.

Future work will include additional rounds involving more complex mixtures and matrices and extension to other measurands that will help improve understanding of the effect of additional parameters that are relevant for these advanced measurement scenarios. With this, the current performance characteristics of NTA can be further determined. This may initiate new developments that remove the current limiting factors. All this would not have been possible without a soundly designed series of round robins. It is envisaged that the challenges to be faced to achieve similar outcomes for biological testing of NP–cell interactions could be even greater, because of the complexity of living organisms and difficulty in controlling such experiments to this level.

Further steps could also be the direct comparison of NTA measurements with other sizing techniques such as measurements based on DLS, which is known to have certain limitations, but is a more established technique.
